# Sustaining Life versus Altering Life-Saving Drugs: Insights to Explain the Paradoxical Effect of Extracorporeal Membrane Oxygenation on Drugs

**DOI:** 10.3390/jcm12113748

**Published:** 2023-05-29

**Authors:** Emna Abidi, Wasim S. El Nekidy, Bassam Atallah, Khaled Al Zaman, Praveen Ghisulal, Rania El Lababidi, Yosef Manla, Ihab Ahmed, Ziad Sadik, Ahmed Taha, Mohamed Askalany, Antoine Cherfan, Mohamed Helal, Saad Sultan, Umar Khan, Vivek Kakar, Jihad Mallat

**Affiliations:** 1 Department of Pharmacy, Cleveland Clinic Abu Dhabi, Abu Dhabi P.O. Box 112412, United Arab Emirates; abidiee@clevelandclinicabudhabi.ae (E.A.);; 2 Cleveland Clinic Lerner, College of Medicine, Cleveland, OH 44195, USA; 3 College of Medicine, University of Sharjah, Sharjah 27272, United Arab Emirates; 4 Critical Care Institute, Cleveland Clinic Abu Dhabi, Abu Dhabi P.O. Box 112412, United Arab Emirates; 5 Heart and Vascular Institute, Cleveland Clinic Abu Dhabi, Abu Dhabi P.O. Box 112412, United Arab Emirates; 6 Faculty of Medicine, Normandy University, UNICAEN, ED 497 Caen, France

**Keywords:** ECMO, antimicrobial drugs, anticoagulant drugs, sedative drugs, analgesic drugs, pharmacokinetics, pharmacodynamics

## Abstract

There has been a substantial increase in the use of extracorporeal membrane oxygenation (ECMO) support in critically ill adults. Understanding the complex changes that could affect drugs’ pharmacokinetics (PK) and pharmacodynamics (PD) is of suitable need. Therefore, critically ill patients on ECMO represent a challenging clinical situation to manage pharmacotherapy. Thus, clinicians’ ability to predict PK and PD alterations within this complex clinical context is fundamental to ensure further optimal and, sometimes, individualized therapeutic plans that balance clinical outcomes with the minimum drug adverse events. Although ECMO remains an irreplaceable extracorporeal technology, and despite the resurgence in its use for respiratory and cardiac failures, especially in the era of the COVID-19 pandemic, scarce data exist on both its effect on the most commonly used drugs and their relative management to achieve the best therapeutic outcomes. The goal of this review is to provide key information about some evidence-based PK alterations of the drugs used in an ECMO setting and their monitoring.

## 1. Introduction

Drug pharmacokinetics (PK) and pharmacodynamics (PD) are subject to many factors, such as patient physiology and drugs’ different physiochemical properties, including protein binding, hydrophilicity and molecular weight, among others. Alterations in drug PK are, in fact, the result of the influence of these properties on its clearance (CL) and volume of distribution (Vd). Patients’ physiology-related alterations occur more frequently in critically ill patients where developed organ insufficiency or failure alter drug elimination rates. A decrease in hepatic perfusion or function can culminate into an induced increase in some drugs’ toxicity. Hydrophilic drugs are mainly affected by both kidney function and blood flow reductions. Moreover, extracorporeal mechanical support (ECMS) has shown additional drug alterations in the same patients, such as an increased Vd in addition to either an increase or a decrease in drug CL [1].

ECMS is a technology used to temporarily replace cardiopulmonary function, including extracorporeal mechanical oxygenation (ECMO), a canonical example [2,3]. ECMO is largely being implemented worldwide as an essential lifesaving technique in many life-threatening situations, such as severe lung damage caused by an infection or cardiogenic shock. The use of ECMO has reached a maximum with the emerging COVID-19 pandemic, and according to the report of the 3rd annual meeting of the Chinese Society of Extracorporeal Support (CSECLS 2019), more than 435 ECMO centers exist worldwide with 13,394 cases treated by ECMO in 2018 [4]. Estimations from the Extracorporeal Life Support Organization (ELSO) report show that the survival rate using ECMO ranges between 58.7% and 73.2% for respiratory support and between 42.7% and 52.6% for circulatory support in a five-year period [5].

The ECMO equipment, mainly formed by a membrane oxygenator and the drive pump, is primarily designed to resolve hypoxemia and restore blood perfusion while the cardiopulmonary system is recovering or during heart–lung transplantation. In an ECMO setting, the pump functions similarly to the heart and drives the blood to flow along the tubes leading out of and flowing into the human body. On the other hand, the oxygenator or “artificial lung” replaces the lung function by ensuring gas exchange as well as regulating temperature [6]. Depending on the indications, ECMO connection to patients with cardiopulmonary failure follows at least two modalities, venous-to-arterial ECMO (VA) and venous-to-venous ECMO (VV) [7].

## 2. ECMO Modalities

### 2.1. VA-ECMO

VA-ECMO is mainly used as a quick and effective life-sustaining ECMS for patients with cardiogenic shock secondary to myocardial infarction or fulminant myocarditis while waiting for recovery or as a bridge to heart transplant [8,9].

### 2.2. VV-ECMO

The use of VV-ECMO generally implies draining the blood from a central vein, e.g., a femoral vein, and then injecting it back into a central vein, e.g., an internal jugular vein, as the most frequent modality [10]. VV-ECMO provides extracorporeal oxygen supply via the oxygenator, leading to improved oxygenated blood supply to the heart and organs with a minimal pulmonary workload. VV-ECMO is largely used in intensive care unit (ICU) patients with reversible lung dysfunction and respiratory failure [11] and can also be indicated in some cases as a bridge to lung transplant [12].

### 2.3. General Effects of ECMO on Drugs

Despite its use as life support for patients with cardiopulmonary failure, ECMO represents a long-duration invasive respiratory and circulatory assisting system. Thus, drug monitoring for ECMO patients is challenging over time, as ECMO affects drugs’ PK at different levels and by different mechanisms [1,13]. Priming solutions that are used to initiate the ECMO support, such as plasma, normal saline, and/or albumin affect mainly hydrophilic drugs’ Vd, leading to significantly decreased drug plasma concentrations and potentially therapy failure. Priming solutions contribute to an increased volume that causes plasma protein dilution, affecting drug–protein binding and leading to supra-therapeutic free drug levels, which may lead to toxicity if the usual drug doses are used, especially with narrow therapeutic index drugs. Drug sequestration is another way that ECMO can cause drug PK alterations due to some properties of ECMO circuits. Sequestration particularly happens within the membrane oxygenator and the circuit tubing due to their large surfaces and affects lipophilic drugs, leading to their adsorption and loss over time [1,14,15].

Antimicrobials, anticoagulants, sedatives, and analgesics are commonly used drugs during ECMO support. Several studies have shown altered PK profiles for these drugs in patients on ECMO [16,17], rendering their effective dosing a challenge to clinicians. However, PK data are still lacking with limited guidance. In this review, we aim to highlight the most important factors that may cause PK profile alterations during ECMO support and discuss possibilities of better pharmacotherapy monitoring.

### 2.4. Alterations in Drug PK Profiles during ECMO

#### 2.4.1. Circuit and Drug Factors

##### Drug Sequestration

Drug sequestration is variable based on both the ECMO setting and the drug in question [1]. Lipophilic and highly protein-bound drugs are more prone to sequestration in ECMO circuits; hence, the relationship between the administered dose and the anticipated blood concentration can be established based on assumptions related to the drug’s physicochemical properties. Generally, these features are reported by the drug’s octanol–water partition coefficient or logP. Moreover, molecular size and drug ionization could theoretically play a role in drug sequestrations [18,19,20].

Although oxygenators provide a large surface area for drug sequestration, research data showed that their contribution is minimal compared to conduit tubing effects [20,21,22]. The drug’s logP determines the drug’s lipophilicity; increased positive logP values indicate augmented lipophilicity, while negative values are proportionally related to decreased lipophilicity. Owing to their relatively higher solubility in the organic component of the ECMO circuit, lipophilic drugs have been constantly shown to be more prone to sequestration when compared with hydrophilic drugs. Additionally, drug sequestration comparisons based on varying degrees of protein binding within ECMO circuits demonstrated that drugs with higher protein binding are found to be considerably sequestrated despite similar lipophilicity [23].

##### Circuit Priming

Different drugs’ PK can be affected by circuit priming. Related influencing factors are the type of priming fluid, pH, temperature, and added electrolytes. The exact phenomenon by which circuit priming may affect drug sequestration, leading to potential consequences such as therapeutic failure or toxicity, is yet to be studied [24], but it could be related to the increased effective circulating volume in ECMO patients following circuit priming.

##### Circuit Age

Circuit type and age can affect the degree of drug loss within the ECMO circuit [14,22,25]. New circuits have shown a higher sequestration effect on specific drugs, such as phenobarbital, vancomycin, gentamicin, and phenytoin, when compared to a used circuit [19]. Other drugs, such as morphine, also showed significantly decreased steady-state concentrations (from 68.2 to 11.6 ng/mL) within new circuits, suggesting that aged circuits could be saturated and lead to less drug sequestration. Thus, in such a situation, the newly used ECMO circuit may require higher drug doses and closer monitoring [19].

##### Patient Factors

Low plasma protein levels, commonly seen in critically ill patients, lead to an increased free fraction of protein-bound drugs, resulting in altered drug CL, Vd, and enhanced drug effects. Moreover, critically ill patients are more prone to significant modifications in serum pH, which may also lead to paralleled dissociation of protein-binding drugs [23]. Vd is also one of the most affected PK parameters in critically ill patients with volume status imbalances and fluid shifts. Furthermore, underlying diseases, associated organ dysfunction, and systemic inflammation would also contribute to multiple PK changes, such as an increase in Vd and a decrease in drug CL [26,27,28]. Other features, such as obesity, may also play a critical role in PK changes. In fact, increased adipose tissue provides sites for the sequestration of lipophilic drugs. Moreover, drugs with lower Vd would have significant alterations in their Vd because of increased patients’ fluid volume compared to drugs with larger Vd, which tend to be lipophilic. Another feature is multi-organ failure, specifically acute kidney injury, a common situation in patients utilizing ECMO, leading to reduced drug CL [29,30].

## 3. Management of Drug Treatments and Outcomes in Patients on ECMO

### 3.1. Anticoagulant Drug Monitoring and Outcomes in Patients on ECMO

Although lifesaving, the ECMO device also presents some complications related to functional events such as device failure. Such events include venous and/or arterial thrombosis as well as hemorrhage, among others. Patients on ECMO support are prone to accelerated risk for thromboembolic events due to sheer stress exposure and blood component interactions with foreign device surfaces of varying biocompatibility [31]. The resulting mechanical forces are at the origin of platelet and coagulation factor activation, fibrinogen deposition, and adherence to device surfaces, followed by thrombin generation, which necessitates the use of anticoagulants. Moreover, hemostasis can be altered because of the patient’s underlying illness, such as cardiogenic shock with liver failure, sepsis-induced coagulopathy, and/or disseminated intravascular coagulopathy. The prevalence of thromboembolic events is between 8.7 to 46.1% [32,33,34]. Reported clot percentages in patients requiring ECMO for respiratory support varies between 13 and 2.7% for oxygenator clots and other clots, respectively. In patients requiring ECMO for cardiac support, the clot percentages are different (9.6 and 0.2%, respectively) [35].

Thus, optimal ECMO anticoagulation is subject to many variables that should be considered simultaneously. Specifically, clinicians should pay closer attention to the patient’s age, underlying illness, duration of ECMO, target antithrombin (AT) activity, and risk of thrombotic or bleeding events. In addition, a set of scheduled diagnostic tests should be performed, including platelet count, AT, activated clotting time (ACT), activated partial thromboplastin time (aPTT), anti-factor Xa (anti-Xa), prothrombin time, and international normalized ratio (INR) [36].

The ELSO guidelines’ (2014) recommendations for adult ECMO patients suggest an ACT of 180–200 s or aPTT of 60–80 s (or 1.5 times the basal value) for patients with low bleeding risk, and an ACT of 160 s or aPTT of 45–60 s for patients at high bleeding risk maintained with a blood flow of >3 L/min. The same flow is recommended for patients with active bleeding with no heparin and not on any anticoagulation. Furthermore, thromboelastography should be performed daily to assess the risk of coagulation [35]. However, different aPTT results can be obtained due to more than 300 laboratory methods used for aPTT monitoring [37]. For example, at a plasma heparin concentration of 0.3 IU/mL measured by factor Xa inhibition, aPTT results can range from 48 to 108 s depending on the reagent used [38]. Thus, the aPTT target range is different from one ECMO center to another and should be based on the type of laboratory method used for monitoring. Dosing examples are summarized in Table 1. Nevertheless, the standard diagnostic tests are limited to guiding the anticoagulation; they are not able to accurately predict clinically relevant hemostasis-related outcomes such as thrombosis or bleeding, rendering a balanced situation hard to achieve [39,40].

Therefore, systemic anticoagulation has been advocated to reduce the risk of thromboembolic events from occurring in the patient and/or the machine’s circuit. On the other hand, the anticoagulation regimen should be closely monitored to assess its efficacy and to ensure patient safety [41,42,43].

#### 3.1.1. Unfractionated Heparin UFH

Unfractionated heparin (UFH) has been universally used as the anticoagulant of choice in patients on ECMO. As of 2019, ELSO recommends a 50–100 unit/kg IV bolus dose of UFH at the time of cannulation, followed by an infusion at 7.5–20 units/kg/h for both VV and VA ECMO [35]. Furthermore, in a meta-analysis, the safety and efficacy in a group of ECMO patients on low-dose anticoagulation (heparin during cannulation only or up to 12,000 ui/24 h, aiming for aPTT < 45 s), compared with another group on a standard dose, revealed that low-dose anticoagulation is a feasible and safe anticoagulation strategy in these patients. Furthermore, fewer associated side effects with the low-dose anticoagulation strategy have been shown. For example, significantly lower rates of gastrointestinal bleeding (OR 0.36, 95% CI 0.20–0.64) and surgical site hemorrhage (OR 0.43, 95% CI 0.20–0.94) were reported in the group of patients who underwent low-dose anticoagulation, while similar rates of hospital mortality and successful weaning off of ECMO were noted in the two groups [44]. However, institutionally based frameworks for anticoagulation monitoring vary between institutions. Examples from different studies [45,46,47] are summarized in Table 1.

#### 3.1.2. Direct Thrombin Inhibitors

Although UFH presents an appealing PK profile characterized by its fast onset, reversibility, close monitoring, wide availability and low cost, direct thrombin inhibitors (DTIs) have also been used as alternatives for patients utilizing ECMO. DTIs have always been chosen, given that they cause relatively fewer heparin-induced thrombocytopenia events or other forms of immune-mediated thrombocytopenia [48]. Besides, the use of DTIs may require fewer circuit exchanges compared to heparin [49]. Moreover, DTIs are associated with further reductions in thrombin level [50]. Furthermore, DTI usage in ECMO settings also refers to their short-acting properties, facilitating their rapid titration to desired anticoagulation levels [51].

Argatroban, a synthetic direct reversible DTI, has demonstrated multiple benefits, as it is fast-acting and achieves a steady state with relatively short half-life. However, its use in the context of adult ECMO settings is not well established [49,52,53]. A systematic review of the available 13 studies, including nine case studies and four cohort studies, included 307 ECMO patients treated with Argatroban. Argatroban was used as a continuous infusion at starting doses varying from 0.05 to 2 μg/kg/min without loading dose in most studies. Doses were titrated to achieve the desired therapeutic target range. Moreover, most of the selected studies used the aPTT as the anticoagulation parameter, as compared to ACT for monitoring purposes. Optimal therapeutic targets varied between 43–70 and 60–100 s for aPTT and between 150–210 and 180–230 s for ACT [54]. To avoid excessive coagulation as well as bleeding complications, aPTT monitoring should be performed 2 h after starting the infusion and after every dosage adjustment until the steady state of aPTT, which is 1.5–3.0 times the initial baseline value to a maximum of 100 s, is established (Table 1) [52,53,55,56,57].

Nevertheless, other alternatives to the aPTT tests, such as the ecarin clotting time or the ecarin chromogenic assay, are preferable in patients who require very high-dose treatment [58]. Argatroban is an alternative for patients with heparin-induced thrombocytopenia (HIT) utilizing ECMO because of its pharmacological properties [51]. The primary elimination route is through the liver; hence, argatroban is the agent of choice in patients with compromised renal function with HIT [53]. On the other hand, argatroban use requires dose adjustment in patients with hepatic impairment and is associated with false elevation of prothrombin time and INR in addition to its high cost.

Bivalirudin, a hirudin analogue, acts by binding to both the active and fibrin-binding sites of thrombin with benefits similar to those of argatroban, such as a short half-life time and use in patients with HIT [59]. Despite limited data and protocols about bivalirudin dosing and monitoring in ECMO, bivalirudin has demonstrated consistent efficacy in this context. The MATRIX trial has demonstrated bivalirudin’s capacity to maintain steady ACT and aPTT values within therapeutic ranges during the complete course of ECMO [60]. Moreover, longer ACT and reaction times were demonstrated for bivalirudin using thromboelastography [61]. However, platelet count and antithrombin activity were not significantly different; bivalirudin reached therapeutic levels faster than UFH (30 vs. 48 h, *p* = 0.03), and these levels were maintained in the therapeutic range more frequently than UFH [62]. Furthermore, significantly higher ACT and aPTT after bivalirudin treatment when compared to UFH treatment have been demonstrated [61].

Moreover, bivalirudin dosing was discussed in a few studies, five of which reported a loading dosage ranging from 0.2 mg/kg to 0.75 mg/kg. Maintenance infusion dosages of bivalirudin also showed inconsistency, ranging from 0.05 mg/kg/h to 1.75 mg/kg/h with an average maintenance rate of 0.27 ± 0.37 mg/kg/h. More specifically, bivalirudin doses in patients were variable between patients with and without continuous renal replacement therapy (CRRT), with values of 0.15 ± 0.06 mg/kg/h vs. 0.28 ± 0.36 mg/kg/h, respectively [63,64,65,66,67] (Table 1). Previous studies reported ACT targets ranging from 160 s to 220 s, while the target of aPTT ranged from 45 s to 80 s. The longest duration of bivalirudin usage was more than 60 days [68,69].

Clinical outcomes of bivalirudin usage in ECMO patients were differently assessed in different studies; platelet count recovery has been reported in several studies [68,70,71], bleeding or thrombosis in other studies [63,68,72,73], mortality in additional studies [63,65,68,71,72,74,75], and the need to change the oxygenator or circuit in 2 studies [55,63]. In specific case scenarios of patients on ECMO with HIT, switching to a bivalirudin anticoagulation regimen was associated with improved platelet counts in a short time and better survival of cases with limited bleeding and thrombotic events [76]. Bivalirudin dosing also requires adjustment in patients with compromised renal function [63].

Finally, both bivalirudin and argatroban showed similarities in achieving and maintaining therapeutic anticoagulation goals, clinical outcomes, and safety in patients with known or suspected HIT. Indeed, Skrupky et al. [77] showed a similar median percentage of aPTT values within the therapeutic range while patients were receiving bivalirudin (92 patients) and argatroban (46 patients) (75% and 70% *p* = 0.238), respectively. However, a greater percentage of aPPT values were supratherapeutic in the argatroban versus bivalirudin groups (18% vs. 8%, *p* = 0.046) [77]. The median IQR DTI dose at the time of first reaching the therapeutic goal was 0.06 mg/kg/hour (0.04–0.08 mg/kg/h) for bivalirudin and 1.0 μg/kg/minute (0.5–2.0 μg/kg/min) for argatroban. The median time to the therapeutic goal was also similar (5.50 [4–14.5] h and 5.75 [3–17.7] h, respectively, *p* = 0.499). Furthermore, new thromboembolic events occurred in 8% of the bivalirudin-receiving group and in 4% of the argatroban-receiving group (*p* = 0.718) [77]. Bleeding events occurred at similar rates in both groups (9% for bivalirudin vs. 11% for argatroban, *p* > 0.999). The total duration of use ranged from 24 to 658 h [77]. In a systematic review and meta-analysis comparing the efficacy of heparin and DTIs during ECMO, both bivalirudin and argatroban showed the best clinical outcomes, especially bivalirudin [78]. On the other hand, a case report showed a failure to respond to bivalirudin, while the same patient showed a fast therapeutic response to argatroban, which can largely be attributed to genetic thrombin mutations as well as to structural defects [79].

#### 3.1.3. Anticoagulation in ECMO and CRRT Patients

Case reports of ECMO patients on CRRT are frequent. In such cases, anticoagulation is subject to many protocols but has not been studied enough. Regional citrate anticoagulation (RCA) is known to be the standard of care [80] for anticoagulation during CRRT, as it has shown much success compared to systemic UFH in terms of filter lifespan and avoiding the effects of systemic anticoagulation as well as due to its associated lower cost [81,82]. Although RCA has been reported to be successful for anticoagulation in simultaneous ECMO and CRRT patients [80,83] (Table 1), its use remains limited, as patients are already on systematic anticoagulation. However, systemic heparinization alone may not prevent higher clotting probabilities in CRRT devices with no heparin coating of tubing and filters. Low blood flow rates during CRRT also cause clotting events in patients on both ECMO and CRTT when compared to patients on ECMO alone [84], which justifies the use RCA as an additional anticoagulant [80]. Furthermore, bivalirudin use in CRRT patients during ECMO has also been documented to be effective, and increasing dosing requirements over the 48 to 120 h after initiation of CRRT have also been described [63] (Table 1).

#### 3.1.4. Anticoagulant-Free ECMO Setting

Although based on limited and small non-randomized studies, a recent systematic review compared the outcomes of anticoagulant-free ECMO to systemic anticoagulated ECMO in 96 patients with VV-ECMO and 58 patients with VA-ECMO and showed no significant differences in the frequency of thrombosis and bleeding events [85]. However, a case report did not support VA-ECMO use without anti-coagulation [86]. Anticoagulant-free ECMO settings were further supported by Robba C et al., who confirmed that the use of ECMO with no initial anticoagulation could be considered a reasonable option, especially in subjects at high risk of bleeding, such as ECMO patients with multi-traumatic injury [87]. In addition, Olson SR et al. hesitantly concluded on the omission of systemic anticoagulation in ECMO patients with active, or at high risk of, bleeding, such as traumatic patients and those with intracranial hemorrhage [85]. Furthermore, results from two large systematic reviews and meta-analyses [88,89] have shown the efficacy of biocompatible surfaces for a cardiopulmonary bypass to achieve major clinical outcomes, such as reducing the incidence of blood transfusion, duration of ventilation, and length of ICU stay. These findings give insights into the use of biocompatible surfaces to mitigate the pro-inflammatory effects of circuit exposure as well as the reduction of platelet activation and adhesion for potential reduction of the risk of adverse bleeding and thrombotic events in the absence of systemic anticoagulation [88,89].

#### 3.1.5. Antiplatelet Therapy

According to current guidelines, a subset of VA-ECMO patients with cardiogenic shock, or after cardiopulmonary resuscitation, must undergo coronary angiography [90,91] and a subsequent percutaneous coronary intervention (PCI) when indicated [92]. Therefore, an indication for dual antiplatelet therapy (DAPT) is probable. A study on a total of 93 patients, from which 26 (28%) were patients with kidney diseases, showed no statistical difference in bleeding in 48 patients on DAPT (51.6%) compared to 42 on no antiplatelet therapy (45.2%), confirming the safe use of DAPT in VA-ECMO patients when indicated [93]. Moreover, a recent study by Baldetti L et al. (2022) [94] assessed the outcomes of a low dose of cangrelor (titrated by ±0.125 μg/kg/min dose adjustments, and up to a maximum of 0.75 μg/kg/min maintenance dose), associated with a standard-intensity anticoagulation with bivalirudin (started at a low dose of 0.01–0.07 mg/kg/h and subsequently adjusted in increments of ±0.02–0.04 mg/kg/h titrated to achieve an aPTT of 50–70 or 45–55) in patients undergoing PCI during VA-ECMO. Coagulation tests were conducted every 6 h. Results showed that the described regimen was a feasible anti-thrombotic strategy in patients receiving PCI and VA-ECMO for acute coronary syndrome-related cardiogenic shock or refractory cardiac arrest [94].

**Table 1:** A selective list of studies highlighting anticoagulant drug monitoring in patients on ECMO and/or RRT.

Study Type	ECLS Modality	Number of Patients	Anticoagulation Approach	Loading Dose	Initial Maintenance Dose	Adjustment to Dosing	Anticoagulation Target
**UFH**							
Multi-center randomized control trial [45]	VV-ECMO	42	Continuous UFH	Bolus 70 or 50 units/kg	18 units/kg/h	12 h after first infusion, heparin was titrated to reach: (a) TEG target (b) aPPT target.	- TEG-K reaction time (R-K): 16–24 min - aPTT ratio: 1.5–2.0 × baseline
Retrospective chart review [46]	VV-ECMO or VA-ECMO	123	Continuous UFH	NA	7 units/kg/h	(a) Supratherapeutic aPTT or TEG: heparin infusion reduced by 100 units/h. (b) Subtherapeutic, aPTT and TEG: heparin infusion increased by 100 units/h. (c) If one value is subtherapeutic and the other therapeutic, the patient’s anti-Xa level is measured.	aPTT: 60–80 s (1.5–2 × baseline) TEG: 2–4 × baseline anti-Xa level of 0.3–0.7 units/mL
Retrospective chart review [47]	VV-ECMO or VA-ECMO	55	UFH sparing; interrupted vs. continuous	Bolus at 100 units/kg	10 units/kg/h or no heparin (≥3 d).	Titrate to ACT target	ACT: 170–230
**DTIs**							
Retrospective chart Review [53]	VV-ECMO or VA-ECMO	9	Argatroban	None	First patient 2 μg/kg/min. Subsequent patients 2 μg/kg/min	Titrate to aPTT target	aPTT: 50–60 s
Retrospective chart review [52]	VV-ECMO or VA-ECMO	10	Argatroban	None	Average initial argatroban dose was 0.175 μg/kg/min	Titrate by 0.05–0.1 μg/kg/min.	aPTT 55–75 s ACT 150–180 s
Case series [57]	VV-ECMO or VA-ECMO	5	Argatroban	None	0.2–2 μg/kg/min	Titrated infusions using incremental changes of 0.05 μg/kg/min every hour to maintain ACTs target.	ACT: 210–230 s
Retrospective cohort study [61]	VV-ECMO or VA-ECMO	13	Bivalirudin	No bolus	Continuous infusion 0.03 to 0.05 mg/kg/h Halved starting dose in patients with reduced creatinine clearance.	Adjusted according to the ACT, aPTT, and *r* time values.	- ACT: 160 to 180 s - Appt: 50 to 80 s TEG *r* time placed at a minimum of 12 and a maximum of 30 min.
Retrospective cohort study [66]	VV-ECMO or VA-ECMO	10	Bivalirudin	No bolus	Continuous infusion of bivalirudin 0.025 mg/kg/h.	When needed, bivalirudin or heparin infusion was increased or decreased step by step, never exceeding 15% of the previous dosage. If a supramaximal aPTT value was recorded, drug infusion was discontinued for 2 h and then started again at a 15% lower dose.	aPTT: 45 to 60 s.
Retrospective study [67]	VV-ECMO or VA-ECMO	44	Bivalirudin	No bolus	Continuous infusion of bivalirudin 0.04 mg/kg/h	Adjusted per aPTT goal.	Low-intensity (45–65 s) or high-intensity (60–80 s)
Single-center, retrospective, observational analysis [63]	VV-ECMO or VA-ECMO	14	Bivalirudin	No bolus (except for one patient, who received 0.2 mg/kg)	0.02 to 0.26 mg/kg/h	Adjusted based on aPTT.	aPTT: 1.5–2.5 times the patient’s baseline. 1.5–2.0 × baseline for those with bleeding concerns
**ECMO patients on CRRT**							
Single-center, retrospective, observational analysis [63]	VV-ECMO or VA-ECMO	4	Bivalirudin for patients on both ECLS and CRRT	No bolus	Median dose of 0.21 mg/kg/h	Adjusted to maintain: aPPT target The median maximum rate patients were titrated to = 0.36 mg/kg/h Individual patients required between 75% and 125% bivalirudin rate increases over the first 48–120 h on CRRT.	aPTT: 1.5–2.5 times the patient’s baseline aPTT. 1.5–2.0 × baseline for those with bleeding concerns
Retrospective chart review [84]	VV-ECMO and simultaneous CRRT (HD mode)	22	UFH and RCA	1—UFH: IV bolus of UFH (50–70 unit/kg) 2—RCA: no bolus	1—UFH continuous heparin infusion (starting at 18 IU/kg/h) 2—RCA: NA	1-a—UFH adjusted during the first 12 h to reach ACT target. 1-b—Heparin infusion is titrated to a target aPTT ratio 2—RCA:NA	ACT: 170–200 s For first 12 h, then aPTT ratio: 1.5 × baseline
Retrospective, single-center study [83]	VV-ECMO and simultaneous CRRT	29	ACDA with or without heparin	Heparin: not mentioned ACD: No bolus	ACDA: at a fixed rate of 240 mL/h	not specified	not specified

ECLS: extracorporeal life support; ECMO: extracorporeal membrane oxygenation; VV-ECMO: venous-to-venous ECMO; VA-ECMO: venous-to-arterial ECMO; UFH: unfractionated heparin; TEG: thromboelastography; aPTT: activated partial thromboplastin time; ACT: activated clotting time; CRRT: continuous renal replacement therapy; HD: hemodialysis; RCA: regional citrate-based anticoagulation; ACDA: anticoagulant citrate dextrose A.

#### 3.1.6. Management of Antimicrobial Drugs in Patients on ECMO

Antimicrobial therapy is essential in sepsis management in critically ill patients to reduce associated mortality. However, dose effectiveness is subject to pathophysiological changes or adjunct therapies such as ECMO support. Thus far, limited data exist as to whether ECMO treatment significantly affects PK/PD parameters in ICU patients. Additionally, data pertaining to the continuous infusion of antibiotics in ECMO patients are scarce. Moreover, no specific dosing recommendations have been formally released. However, primary data pointed toward alterations in antibiotic serum concentrations, while standard dose regimens might still be effective [95]. On the other hand, subtherapeutic and supratherapeutic free drug concentrations of antimicrobial agents have recently been reported to be associated with failure of therapy or expressing adverse events, respectively [96]. To the best of our knowledge, scarce data exist about dosing recommendations in critically ill patients on ECMO to counterbalance the PK alterations caused by the underlying illness or by drug sequestration in the ECMO circuit [97].

The therapeutic drug monitoring (TDM) study performed on ECMO patients by Kühn et al., in 2020, compared antibiotic serum concentrations in patients with and without ECMO support [98]. A total of 112 antibiotic total drug serum concentration measurements from patients on ECMO support and 186 samples from non-ECMO patients were analyzed. Results showed significantly lower median serum concentrations for piperacillin (32.3 vs. 52.9; *p* = 0.029) and standard dose of meropenem (15.0 vs. 17.8; *p* = 0.020) in the ECMO group. However, both groups had similar concentrations for ceftazidime, high-dose meropenem (6 g/day), and linezolid. Moreover, this study also showed that pre-specified target serum concentrations for piperacillin and linezolid were not reached in 48% and 35% of patients on ECMO, respectively, compared to ≤35% and ≤20%, respectively, in the non-ECMO group. In addition, after adjustment for age, sex, body mass index, and renal function to the influence of multiple clinical factors on antibiotic serum concentrations, ECMO support was independently associated with reduced serum concentrations of both piperacillin and the standard dose of meropenem. However, ECMO blood flow rate was not a factor that affected the decreased concentrations of these antibiotics. On the other hand, the duration of ECMO membrane oxygenator use was associated with increased serum concentration of most antibiotics [98]. Moreover, the same study evaluated the microbiological cure in some of the studied patients. Results showed the detection of a total of 89 potential bacterial pathogens in clinical specimens of 17 patients on ECMO support and 26 in non-ECMO patients. Respiratory infections were the most detected (50/89, 56.2%), followed by bloodstream infections (26/89, 29.2%), while urinary tract or soft tissue infections were less reported (13/89, 14.6%), respectively. Gram-negative bacteria were often detected (64/89, 71.9%), with *Escherichia coli* (*n* = 22), *Pseudomonas aeruginosa* (*n* = 13), *Klebsiella pneumoniae* (*n* = 9), and *Enterobacter cloacae* complex (*n* = 7) the most frequently detected. *Staphylococcus aureus* was the only relevant Gram-positive bacterium recovered from respiratory specimens, whereas coagulase-negative *staphylococci* and *enterococci* were mainly seen in blood cultures. Most studied pathogens were susceptible to the investigated antibiotics, with median minimum inhibitory concentrations (MICs) below the respective EUCAST breakpoints [98]. It is worth mentioning that investigators in this study did not check free drug concentrations, which could be affected by all the previously mentioned factors in patients utilizing ECMO.

Contrarily, a recently published meta-analysis demonstrated that the impact of ECMO support on hydrophilic drugs, such as piperacillin and meropenem, appears to be negligible [97]. Furthermore, intermittent infusion of piperacillin and meropenem resulted in no differences in serum concentrations between 26 patients who utilized ECMO compared to 41 matched controls in a TDM study by Donadello et al. in 2015 [95]. However, more than 60% in both patient groups did not reach the adequate target concentrations (free drug concentration of 4–6 times higher than the respective MIC breakpoint). In another TDM study, Hanberg and al. examined the effects of intermittent infusion of meropenem in patients on ECMO support; results showed that a standard dosing regimen (1 g IV every 8 h) did not achieve a free drug serum concentration above the MIC of Gram-negative pathogens for the entire dosing interval, pointing to the need for substitute dosing strategies in critically ill patients on ECMO where higher odds of multi-resistant pathogens have been consistently demonstrated [99].

The above-mentioned observations might not have accounted for the drug sequestration in the ECMO circuit due to its wide surface. This has been demonstrated in both an ex vivo and an in vivo ovine model study for lipophilic and highly protein-bound antibiotics [100,101]. Additionally, increased ceftazidime and meropenem serum levels could have been due to prolonged use of the same ECMO membrane oxygenator (membrane vintage). More device-related situations, such as a capillary leak, substantial fluid shifts, and the amplified distribution volume in critically ill patients, might also be the reasons behind altered antibiotic serum concentrations. A positive fluid balance has always been discouraged in those patients [102]. The PK of antibiotic, antiviral, antituberculosis, and antifungal agents are prone to severe changes in patients on ECMO support. Here, we discuss the most administered agents. Table 2 summarizes the pharmacokinetic changes for antimicrobials during ECMO.

## 4. Aminoglycoside Antibiotics

### 4.1. Amikacin

A case–control study comparing critically ill patients on ECMO support with a matched group without ECMO reported no significant differences in amikacin PK [103]. Generally, it is recommended to dose aminoglycosides with close therapeutic drug monitoring (Table 2).

#### 4.1.1. Beta-Lactam (Carbapenem) Antibiotics

##### Imipenem

Limited data in this context were restricted to a study demonstrating a high disparity in concentrations between two ECMO-supported patients (11.3 and 2.7 mg/L) [104]. Another study showed that pharmacological targets against less susceptible pathogens may require higher dosage in critically ill patients on ECMO [105] (Table 2).

##### Meropenem

A case-matched control study showed that continuous infusion of meropenem resulted in a significant decrease in CL (7.9 L/h vs. 11.7 L/h), while a non-significant increase was noted in the drug Vd (0.45 vs. 0.41 L/kg) [95]. Being renally cleared, meropenem dosing remains subject to modifications relative to specific clinical scenarios that are essentially related to the patient’s renal function. Sensibility to external circuit temperature might also affect the drug integrity, as meropenem degradation occurs at 37 °C [95,106] (Table 2).

##### Piperacillin/Tazobactam

Patients on ECMO support have shown non-significant increases in CL and Vd (156 mL/min and 0.33 L/kg, respectively) when compared to their non-ECMO counterparts (134 mL/min and 0.31 L/kg, respectively) [95]. A study by Cheng V et al. suggests that the PK of piperacillin and tazobactam are not significantly affected by the introduction of ECMO. The same study results show that the dosing of piperacillin and tazobactam should be guided by CrCL, BMI, and the presence of RRT as per dosing standard recommendations for critically ill patients not on ECMO [107] (Table 2). Nevertheless, the 13.5 g piperacillin/tazobactam dosage regimen (divided into three or four applications per day or administered as a continuous infusion) is still the most utilized on a worldwide basis and is also supported by the Surviving Sepsis Campaign [94,108].

#### 4.1.2. Glycopeptide Antibiotics

##### Vancomycin

Limited data exist on vancomycin PK changes during ECMO. Indeed, two studies reported no statistically significant differences in the Vd, CL, or elimination rate constant (KE) between ECMO receivers and non-receivers among adult patients [109,110]. However, concomitant use of albumin might have prevented vancomycin from binding within the ECMO circuit, leading to a non-significant loss of the drug, which was observed in the study [110] (Table 2). Furthermore, another study conducted on adults also demonstrated a longer mean time to reach the target vancomycin trough concentration in the ECMO group compared to the non-ECMO one [111].

Furthermore, Marella P et al. evaluated TDM-guided vancomycin dosing effectiveness in adult patients on ECMO [111]. In this study, the average percentage of measurements in the therapeutic range was 24%, with 46% subtherapeutic and 30% supratherapeutic. The same study showed that patients on ECMO are more likely to have subtherapeutic vancomycin concentrations in the early phase and that treating individuals undergoing simultaneous renal replacement therapy requires vigilance. To assess treatment effectiveness in critically sick patients on ECMO, the authors recommended a TDM-based vancomycin dosage [111]. Several studies evaluating adults’ PK data suggest that ECMO has little effect on vancomycin Vd and CL [109,110,112]. The use of ECMO priming fluids, transfusion and hemodilution, simultaneous administration of nephrotoxic medications, and decreased renal function are all explanations for altered vancomycin PK during ECMO [1]. For instance, Park et al. assessed the efficacy of a vancomycin dosage method based on total body weight and creatinine clearance in adult patients on ECMO and showed that a dosing strategy of 15 to 20 mg/kg/dose every 8 to 12 h is not sufficient in most of those patients to achieve the target trough in the initial period [109].

#### 4.1.3. Macrolide Antibiotics

##### Azithromycin

Pharmacokinetic parameters in three adults with acute respiratory distress syndrome utilizing ECMO and treated with azithromycin were compared to documented data from patients without ECMO. Results showed no differences in maximum and minimum concentrations, the area under the curve (AUC), and CL between both groups, while Vd was decreased in the ECMO-supported group. General observations showed no considerable effects on either plasma concentrations or azithromycin concentrations at the infection site in patients on ECMO support [113] (Table 2).

#### 4.1.4. Neuraminidase Inhibitors

##### Oseltamivir

A single prospective population study compared the PK parameters of oseltamivir carboxylate in 14 adults utilizing ECMO to the PK values of healthy volunteers. Results demonstrated a significant increase in Vd (179 vs. 26 L) during ECMO [114] (Table 2). Owing to its renal clearance and the decreased renal function in ECMO patients, the mean CL of oseltamivir carboxylate was significantly reduced when healthy volunteers were compared to ECMO patients with substantially decreased renal function [114,115]. Furthermore, patients on ECMO often may develop acute kidney injury, which might affect drug elimination, with higher C_max_ and AUC than patients on ECMO support alone with normal kidney function [116] (Table 2). Considerable differences in oseltamivir concentrations between pre- and post-ECMO oxygenator membranes were not observed, eliminating the possibility of oxygenator drug binding in this context [20]. Thus, dose adjustment for ECMO is not required unless there is a concomitant kidney dysfunction. The main adverse events documented were limited to gastrointestinal issues [114].

#### 4.1.5. Oxazolidinone Antibiotics

##### Linezolid

Linezolid dosing has been particularly challenging in the context of an ECMO setting. Moreover, while limited data exist on linezolid use in those patients, intermittent infusion of 600 mg of linezolid every 12 h in three ECMO patients did not achieve clinically effective serum concentrations in a case-series study [117] (Table 2).

#### 4.1.6. Antifungal Agents

Candida infections are common and challenging to treat in patients on ECMO support [118]. A single case report discussed the PK of voriconazole and caspofungin during ECMO. Voriconazole showed decreased CL (49.33 vs. 140 mL/min), no changes in Vd (1.38 vs. 1.39 L/kg), and a higher mean peak concentration (13.91 vs. 5.4 μg/mL) when compared to reference values [119] (Table 2). PK changes should be attributed partially to the patient’s defective intrinsic metabolism and, in this specific study, to the sequestration of the drug on the ECMO circuit at earlier phases. However, ECMO-related PK changes of voriconazole, a time-dependent antifungal agent, are to be investigated in these patients. Indeed, determining the optimal dosing regimen during ECMO is crucial [120] since some fungal infections are difficult to treat due to their adherence to the circuit’s indwelling catheters, rendering their eradication complicated. On the other hand, the PK parameters of caspofungin showed no significant changes [119]. Optimal levels were possible with standard dosing in adult patients on ECMO support, as the drug was not sequestrated in the circuit [119].

#### 4.1.7. Antituberculosis Agents

Isoniazid, rifampicin, ethambutol, and pyrazinamide are among the standard first-line antituberculosis agents. Based on two case reports of adults utilizing ECMO, the antituberculosis agents were affected by different aspects. However, the reported ECMO patient on rifampicin and ethambutol was also on concomitant and extended dialysis modality, which made results difficult to interpret. However, their elimination by the ECMO membrane was not perceived when pre- and post-ECMO filter values were measured [121] (Table 2). A second case report showed rifampicin plasma concentration to be below the therapeutic level, regardless of the initial use of a higher dose of 750 mg [122]. This confirmed that rifampicin PK are subject to either ECMO-related or patient-related variations. Variations in the drug’s PK might also be explained by its lipophilic nature leading to potential sequestration within the ECMO circuit and ultimately affecting patients’ required doses. Subtherapeutic concentrations lead to underexposure to rifampicin in this population and would also be attributed to tuberculosis-induced inflammation and an associated increase in CYP450 leading ultimately to increased drug metabolism [122] (Table 2).

### 4.2. Management of Sedation and Analgesia in Patients on ECMO

Paradigm shifts in analgosedation regimens and PK alterations of commonly used analgesics and sedatives have been reported in ECMO patients [123]. Limited data exist on the most appropriate opioids and sedatives in critically ill patients utilizing ECMO to achieve the desired level of sedation while minimizing excess exposure. Many sedatives and analgesics, such as fentanyl, benzodiazepines, and propofol, present with a high potential for sequestration within the ECMO circuit, secondary to their lipophilic nature. As a result, initial underdosing of these classes is expected in this population [96]. Of note, with prolonged use of these agents in the ECMO circuit, the saturated membranes may act as delayed-release reservoirs at a time when the aim is to wean patients off these agents, leading to prolonged sedation. Furthermore, patient-related factors such as acute kidney injury, increased cardiac output and increased circulating blood volume during ECMO imply additional changes in some patients’ sedation requirements [96]. Using analgosedation has been a common approach to manage critically ill patients utilizing ECMO [124,125]. Adults receiving ECMO for respiratory failure appear to have increased requirements for analgesia and sedation over time [126,127]. The first case report demonstrating increased sedation requirements was in a 30-year-old man with severe respiratory failure requiring veno-venous ECMO as a bridge to lung transplantation. The morphine and propofol maintenance doses to maintain appropriate levels of sedation significantly increased over 19 days while utilizing ECMO support [126].

#### 4.2.1. Sedation

While sedation represents a key element in the overall management of patients on ECMO, limited studies describe sedation in this context. Its management represents a challenge, given the need for higher dosing frequently observed in ECMO patients [127,128]. Sequestration, adsorption, and loss in the ECMO circuit are the most common conditions affecting sedative dosing, such as with midazolam and propofol, in critically ill patients utilizing ECMO [16,23,129,130]. In this situation, higher doses are usually needed to maintain sedation compared to non-ECMO critically ill patients [127,128]. Propofol, with its fast onset and offset actions, short-terminal half-life, minimal active metabolites, and lack of increased delirium risks, made it the first-line sedating agent employed across different cohorts of critically ill patients (i.e., medical, surgical, or neurological) [131]. However, neither dosing nor safety profiles have been assessed yet in the context of an extended ECMO setting. Results from the first retrospective analysis of real ECMO patient data confirmed the possible safety of propofol in the absence of an increased risk of oxygenator failure [132]. The same results were also confirmed in a subsequent study by Lamm et al. [133].

Dexmedetomidine, a central acting α-2 agonist with lesser lipophilic extent compared to propofol, constitutes an attractive option for general sedation in the critically ill patient population [22]. Pharmacokinetic changes in ECMO have only been studied in vitro, showing a significant loss in the ECMO circuitry that has been related to adsorption to polyvinyl chloride tubing. Nevertheless, results concerning the clinical effect of the described changes have not yet been reported [22]. Data on alterations of the PK of phenobarbital, a long-acting barbituric acid derivative, are limited to a small amount of data on neonates demonstrating increased drug dosing necessities associated with the duration of ECMO time, where longer-term use was associated with an increase in drug adsorption and an increased probability of therapeutic failure [134,135].

Furthermore, a maximum of 50% drug loss in ECMO circuitry has been widely reported with benzodiazepines and specifically midazolam. Indeed, significant increases in midazolam requirements also have been reported [127,136] (Table 3). Other benzodiazepines have not been evaluated for sedation management in the ECMO population. However, in vitro, data showed controversy about lorazepam, as in a few studies, the drug showed the least risk of adsorption to ECMO circuitry and overall drug loss, while in other studies, there was a substantial extent of drug loss [16,124]. The greatest amount of drug loss in ECMO circuitry has been observed with diazepam because of its lipophilicity; the same drug has also been associated with accumulation of active metabolites.

**Table 2:** A selective list of studies highlighting antimicrobial drug monitoring in patients on ECMO and/or RRT.

Antimicrobial/Study Type	ECLS Modality	Number of Patients	Reported PK Parameter	Dose	Study-Specific Recommendations
**Aminoglycoside antibiotics**					
**Amikacin** **Case–control study** [103]	VV or VA-ECMO	46	**C_max_ (mg/L):** 71.7 (58.9–79.7) C_max_ < 60 mg/L in 26% C_max_ > 80 mg/L in 24% **AUC (mg.h/mL):** 973 (799–1193) **C_min_ (mg/L):** 8.5 (3.0–15.4)	15–20 mg/kg doses, interval by TDM.	- Therapy should align with dosing strategies commonly used in critically ill patients not receiving ECMO therapy. - High-dose, extended-interval strategies are recommended. - TDM is recommended. - For those with compromised renal function or utilizing RRT, dose per levels.
**Beta-lactam (carbapenem) antibiotics**					
**Imipenem** **Case Series** [104] **Case Series** [105]	VV-ECMO VV or VA-ECMO	2 10	**MIC mg/L:** 0.125 and 0.25. **Vd (L):** 13.98 **CL (L/h):** 9.78	1 g q6 h. 0.5 g q6 h	- Therapy should align with dosing strategies commonly used in critically ill patients not receiving ECMO therapy. - An elevated dosing regimen (4 g/24 h) is more likely to optimize drug exposure. - Administration should be by either an extended infusion or continuous infusion strategy.
**Meropenem** **1-Case–control study** [95] **2-Matched cohort study** [106]	VV or VA-ECMO (9 on CRRT) VV-ECMO or VA-ECMO (3 on CRRT)	26 14	Vd (L/kg): 0.46 (0.26–0.92) t ½ (h): 3.0 (2.1–4.8) CL (mL/min): 125 (63–198) **Vd (L/kg):** 29.7 ± 19.2 ve **CL (mL/min):** 17.4 ± 14.8 L/h	At Cr CL of: a: >80 mL/min: 1 g q8 h b: 51–80 mL/min: 1 g q12 h c: 10–50 mL/min: 0.5 g q12 h d: <10 mL/min: 0.5 g daily -CRRT: 1 g q8 h 1 g (IV) bolus and 1 g IV q8 h	- Therapy should align with dosing strategies commonly used in critically ill patients not receiving ECMO therapy. - Administration should be by either an extended infusion or continuous infusion strategy unless patients have compromised kidney function or are utilizing RRT. - No significant influence of ECMO on PK. - Highly variable PK parameters in patients with sepsis. - High proportion of patients not achieving target concentrations. - Target attainment of meropenem is poor under standard dosing in critically ill patients but is not influenced by ECMO. - No additional dose in the RRT-dependent patients post RRT.
**Piperacillin/tazobactam: 1-Case control study** [95] **Prospective, open-labeled, multicenter PK study** [107]	VV-ECMO or VA-ECMO (9 on CRRT) VV and VA-ECMO (14 on RRT)	14 27	**Vd (L/kg):** 0.33 (0.26–0.46) ***t*_1/2_ (h):** 2.0 (1.1–4.2) **CL (mL/min):** 156 (91–213) **Vd (L/kg):** 0.51 **CL (L/h):** 12.02	At Cr CL of a: >80 mL/min: 4.5 g q6 h b: 51–80 mL/min: 4.5 g q6 h c: 10–50 mL/min: 4.5 g q6 h d <10 mL/min: 4.5 g q6 h -CRRT: 4.5 g q6 h 4.5 g LD, then 4.5 g q6–8 h	- No significant influence of ECMO on PK. - Therapy should align with dosing strategies commonly used in critically ill patients not receiving ECMO therapy. - Administration should be by either an extended infusion or continuous infusion strategy. - Dosing should be guided by CrCL, BMI, and the presence of RRT as per dosing standard recommendations for critically ill patients not on ECMO. - Administration should be by either an extended infusion or continuous infusion strategy.
**Glycopeptide antibiotics**					
**Vancomycin** **Retrospective study** [109] **Prospective, matched cohort, single center, pharmacokinetic study** [110]	1-VV and VA-ECMO	20 11	**K (h^−1)^:** 0.12 ± 0.04 **CL (L/h):** 4.62 **Vd (L/kg):** 0.65 **K (h^−1^):** 0.088 ((0.055)) **Vd (L/kg):** 0.84 (0.24)	Total daily dose: 32.54 mg/kg q2.10 ± 0.72/day. Initial dose: 15–25 mg/kg Maintenance dose: calculated to achieve to achieve trough levels within 10–20 mg/L.	- Similar elimination rate with non ECMO patients. - Dosing strategy not sufficient to achieve the target trough in the initial period in most patients receiving ECMO. - TDM is recommended. - Therapy should align with dosing strategies commonly used in critically ill patients not receiving ECMO therapy. - Clearance in ECMO patients with a roller pump was significantly lower than that in the matched cohort. - PK parameters in ECMO patients with a centrifugal pump were comparable to those in the matched control group.
**Macrolide antibiotics**					
**Azithromycin** [114]	VV-ECMO	3	**C_max_ (mg/L):** 4.0 ± 0.5. **C_24_ (mg/L):** 0.22 ± 0.1 **AUC_0–24_ (mg-h/L):** 9.8 ± 2.6 **CL (mL/min/kg):** 8.0 ± 4.9 **Vd (L/kg):** 19.8 ± 7.6	-IV infusion of 500 mg q24 h	- No significant influence of ECMO on plasma concentrations.
**Neuraminidase inhibitors**					
**Oseltamivir** [115] **Single-center, prospective, open-label, population PK study** [117]	VV-ECMO (4 on CVVHF) VV-ECMO (3 on concomitant CVVHF)	14 7	**CL (L/h):** 15.8 (4.8–36.6) **Vd (L):** 179 (61–436) AUC (ng/hour/mL): 4346 (644–13,660) **C_max_ (ng/mL):** 509 (54–1277) -Patients with preserved renal function: **C_max_ (ng/mL):** 1029 ± 478 **AUC (mcg/h/mL):** 9.00 ± 4.52 -Patients on ECMO and CVVHF: 4- to 5-fold higher C_max_ and AUC	75 mg twice daily 75 or 150 mg twice daily	- Dosage adjustment for ECMO, per se, appears not to be necessary. - Doses should be reduced in patients with renal dysfunction. - No significant influence of ECMO on PK. - Drug accumulation in the plasma of patients on ECMO plus CVVHF for renal failure. - Drug dosage should be decreased, and plasma levels of drug should be monitored in patients receiving CVVHF because of acute kidney injury.
**Oxazolidinone antibiotics**					
**Linezolid** **case-series study** [118]	NA	3	**C_max_ (mg/L):** 15.67, 18.51 and 15.61. **Cmin (mg/L):** 4.25, 0.47 and 0.43. **AUC0–24 (mg h/L):** 212.58, 165.65 and 100.59. **CL (L/h):** 5.65 7.24 13.35 **Vd (L):** 49.7, 17.6 and 46.77 ***t*_1/2_ (h):** 6.10, 1.68 and 2.20	Infusion: 600 mg q12 h	NA - PK targets are not achieved with standard dosage of linezolid when the MRSA MIC is >1 mg/L. - Patients with *S. aureus* infection and MICs > 1 mg/L on ECMO might be at considerable risk of ineffective PK. - Increased dosage as well as prolonged or continuous infusion of linezolid might be considered to increase AUC0–24/MIC ratios or t > MIC. - Future studies to confirm the above recommendations are necessary.
**Antifungal agents**					
**Voriconazole and caspofungin** **Case series study** [120]	VV-ECMO	2	**Caspofungin:** -**Mean trough (μg/mL):** 3.73 -**mean peak (μg/mL):** 11.95 ***-t*****_1/2_ (h):** 13.60 ***-Vd*** **(L):** 8.22 **-CL (mL/min):** 6.90 **Variconazole:** -**Mean trough (μg/mL):** 9.65 -**Mean peak (μg/mL):** 13.91 ***-t*****_1/2_ (h):** 21 -***Vd*** **(L):** 1.38 **-CL (mL/min):** 49.33	**Caspofungin:** loading dose: 70 mg/day maintenance dose: 70 mg once daily. **Variconazole:** IV loading dose: 400 mg twice daily maintenance dose: 280 mg twice daily.	- Adequate caspofungin plasma levels are maintained during ECMO. - It is recommended to monitor variconazole: plasma levels to ensure efficacy and avoid toxicity.
**Antituberculosis agents**					
**Ethambutol and rifampicin** **Case report** [122]	VV-ECMO/extended dialysis	1	Dialyzer clearance: **Ethambutol:** Whole blood: 1 mL/min (range 51–131 mL/min) Plasma: 95 mL/min **Rifampicin:** Whole blood: Between 53 and 77 mL/min Plasma: Between 39 and 53 mL/min.	- IV Ethambutol: 1000 mg/day - IV rifampicin: (600 mg/day)	- TDM should be used to guide Ethambutol dosing in patients undergoing extended daily dialysis.
**Ethambutol/rifampicin and Pyrazinamide** [123]	VV-ECMO	1	**Rifampicin:** Serum **C_max_** (μg/mL): 8–24 Serum **T_max_** (h): 0.75–2/ **Ethambutol:** Serum **C_max_** (μg/mL): 2–6 Serum **T_max_** (h): 2–3 **Pyrazinaminde:** Serum **C_max_** (μg/mL): 20–50 Serum **T_max_** (h): 1–2	Rifampicin: 750 to 1200 mg/day Ethambutol: 1200 to 1600 mg/day Pyrazinaminde: IV: 1200 to 1600	- TDM is needed to achieve appropriate target concentrations for antituberculosis agents in patients with miliary tuberculosis under ECMO.

ECLS: extracorporeal life support; ECMO: extracorporeal membrane oxygenation; VV-ECMO: venous-to-venous ECMO; VA-ECMO: venous-to-arterial ECMO; AUC: area under the curve; TDM: therapeutic drug monitoring; RRT: renal replacement therapy; MIC: minimum inhibitory concentration; Vd: volume of distribution; CL: clearance; CRRT: continuous renal replacement therapy; CrCL: creatinine clearance; PK: pharmacokinetics; IV: intravenous; BMI: body mass index; CVVHF: continuous venous-to-venous hemofiltration; *t*_1/2_: drug half-life; MRSA: methicillin resistant staphylococcus aureus.

#### 4.2.2. Analgesia

##### Parenteral Opioid Analgesia

Because of their distinguished PK and PD, parenteral opioids represent the essential analgesics in the context of critically ill patients, specifically for those utilizing ECMO. Remifentanil has a rapid onset and short duration of action, attributable to its significantly increased lipophilicity and non-renal elimination pathways [137].

Moreover, fentanyl-based sedation also showed numerically lower requirements for non-analgesic sedatives and a statistically significant reduction in benzodiazepine requirements [138].

However, fentanyl use in the setting of obese ECMO patients is at risk of drug accumulation followed by a possible subsequent depot/reservoir effect, which may lead to prolonged respiratory depression [139,140]. The high lipophilicity increases apprehensions about the drug adsorption to ECMO circuit tubing. Indeed, lower fentanyl equivalent dose requirements and more delirium-free and coma-free days were observed with hydromorphone compared to fentanyl-based sedation in adult patients on ECMO support in a retrospective propensity-matched analysis study [141] (Table 3).

On the other hand, hydromorphone undergoes hepatic metabolism to inactive metabolites that are subsequently renally eliminated [142]. However, morphine is metabolized via the liver to active metabolites that accumulate in patients with renal issues. Thus, despite its advantageous lower extent of lipophilicity, its use in the setting of renal impairment is less favorable compared to fentanyl, especially in the context of initial analgesia where the accumulation of active metabolite can lead to non-desired deeper levels of sedation. Finally, we suggest that morphine and hydromorphone may serve as useful analgesic alternatives in patients with uncontrolled pain receiving ECMO because both agents are more hydrophilic than fentanyl [143].

Sufentanil is a synthetic opioid drug with a rapid onset and is more potent (5 to 10 times) than fentanyl [144]. It is highly protein bound and excreted as metabolites in the urine (80% metabolites) after being metabolized in the liver [145]. A wide range of variability in sufentanil PK is expected in ECMO patients [146]; however, scarce data are available on these patients. In a prospective PK study that included 20 patients who received sufentanil during VA-ECMO, the authors found that based on Monte Carlo simulation, an infusion of 17.5 μg/h appeared to achieve target sufentanil concentrations (0.3–0.6 μg/L) in most ECMO patients, excluding hypothermic patients (33 °C). In these hypothermic patients, over-sedation with the potential of inducing respiratory distress should be monitored, particularly when their plasma protein level is low [147].

Furthermore, it has been largely demonstrated that parenteral opioids can be quickly titrated down because of their short half-life. An example is methadone, described by its physicochemical properties, including hydrophilicity and extent of protein binding, rendering its use favorable in ECMO patients [148]. Methadone, at 30 mg intravenous four times a day/40 mg by mouth four times a day in one case and at 10 mg intravenous three times a day/40 mg by mouth three times a day in a second case of prolonged VV-ECMO, was used as an adjunct to wean off commonly used sedation agents in an ECMO setting; protocol examples were described by Dong E et al. [148] (Table 3).

#### 4.2.3. Nonopioid Analgesia

Data about using non-opioid analgesic agents such as ketamine in patients on ECMO support are scarce. Consideration of adjunct agents, such as sub-anesthetic doses of ketamine, may facilitate achieving sedation goals. Two uncontrolled studies demonstrated reductions in sedative rates with the addition of low-dose ketamine infusions [149,150]. However, most recently, a randomized trial did not show any differences in opioid or sedative requirements with the addition of low-dose ketamine to standard sedation practices as compared to standard sedation practices alone in patients receiving VV-ECMO for severe respiratory failure [151]. The median cumulative amount of fentanyl and midazolam equivalents in the low-dose ketamine group were almost twice and four times as high, respectively, compared to the control group from ECMO initiation to the decision to achieve wakefulness [151]. However, patients receiving low-dose ketamine infusion had similar improvements in their RASS scores over the 72 h after the decision to achieve wakefulness [151] (Table 3).

**Table 3:** A selective list of studies highlighting sedative and analgesic monitoring in patients on ECMO and/or RRT.

Sedative/Study Type	ECLS Modality	Number of Patients	Dose	Recommendations
Midazolam -Retrospective chart review [128]	VV or VA-ECMO and RRT	29	- Median daily dose: 175 mg (range 24 to 1092 mg)	- A significant increase for midazolam in patients on ECMO is needed. - Patients on VV-ECMO need higher doses than patients on VA-ECMO. - No significant influence of RRT on average doses over time (one to 21 days) for midazolam in both VA and VV groups.
-Retrospective comparative cohort analysis [129] -Benzodiazepines/prospective, observational study [137]	VV or VA-ECMO (11 on RRT) VV or VA-ECMO	34 32	- All sedatives were converted to midazolam equivalents: midazolam 5 mg/h = lorazepam 3 mg/h = propofol 200 mg/h = dexmedetomidine 74.1 μg/h. - Benzodiazepines were converted to midazolam equivalents: 1 mg IV lorazepam = 3 mg IV midazolam = 5 mg IV diazepam.	- Amount of sedation nearly twice as high in ECMO patients. - No increase in dose requirement over time during ECMO. - VV-ECMO patients had a higher median dose of opioids and almost a lower dose of benzodiazepines than VA-ECMO patients. - Delirium was more commonly identified in the VV group.
-Opioids/prospective, observational study [137] -Hydromorphone and fentanyl/single-center retrospective observational study [139]	VV or VA-ECMO VV or VA-ECMO	32 148	- Opioids were converted to fentanyl equivalents: 200 μg IV fentanyl = 1.5 mg IV hydromorphone = 10 mg IV morphine. - IV-fentanyl equivalents: 0.1 mg IV fentanyl = 1.5 mg IV hydromorphone. - Oral dosage forms were considered with hydromorphone 1 mg oral (PO) being equivalent to fentanyl IV 13.3 µg.	- No increase in dose requirement over time during ECMO. - More days alive without delirium or coma while significantly reducing narcotic requirements with hydromorphone-based sedation in ECMO patients.
Methadone/case series study [148]	VV-ECMO	2	Case 1: 30 mg intravenous 4 times a day/40 mg by mouth 4 times a day Case 2: 10 mg intravenous 3 times a day/40 mg by mouth 3 times a day)	Effectiveness was demonstrated by decreasing other opiates and sedatives without the need for dose escalation in this population.
Ketamine -Case series [149] -A randomized controlled trial [151]	VV or VA-ECMO 2-VV-ECMO	26 10	- Median starting infusion rate: 50 mg/h - NA	- Ketamine infusion can be used as an adjunctive sedative agent in patients receiving ECMO. - Decreased concurrent sedative and/or opioid infusions without altering RASS scores. - Addition of ketamine to standard sedation protocol does not reduce use of sedatives or opioids.

ECLS: extracorporeal life support; ECMO: extracorporeal membrane oxygenation; VV-ECMO: venous-to-venous ECMO; VA-ECMO: venous-to-arterial ECMO; RRT: renal replacement therapy; IV: intravenous.

## 5. Discussion

Drug interactions in ECMO settings have been demonstrated in adult critically ill patients. However, failure to understand the impact of ECMO on some drugs’ PK leads to successive therapeutic failures and/or drug toxicity. Therefore, focused efforts have been demonstrated to prevent thrombotic complications that could happen within the ECMO circuit and to avoid bleeding in the same patient. However, the frequency of both events remains high. Difficulties in managing anticoagulation in this patient cohort are primarily attributed to the infinite complexity of critically ill patients’ situations, especially those on ECMO.

Contexts of optimal pharmacotherapy management in critically ill patients also reveal more challenges in antimicrobial dosing while on ECMO support. Constraints can be due to complex pathophysiological alterations meaningfully impacting many antimicrobial PK/PD characteristics. Furthermore, some limitations are also related to serum drug concentration measurements, as total antibiotic serum concentrations instead of free unbound drug fractions are more often measured [98]. This leads to an overestimation of the actual amount of effective antibiotic substance in the patient’s bloodstream, as the protein-bound fraction of antibiotics is pharmacologically inactive.

An example is the piperacillin protein-bound fraction, which is estimated to reach 20–30% of the total serum concentrations in ICU patients [152], rendering the "real" risk of piperacillin/tazobactam underdosing higher than reported in some studies [98]. Thus, accounting for a protein-bound fraction of multiple antibiotics would have made the resulting serum concentrations proportionally more or less sufficient in both non-ECMO and ECMO patients. Finally, it is also of major importance to verify, in many research contexts of critically ill patients, whether the antibiotics in question might have been clinically effective in some cases with serum concentrations below the pre-specified target, as the measured pathogen MICs could have been, in many cases, different than the respective EUCAST breakpoints [98].

Results from the Analgesia, Sedation, and Antibiotic Pharmacokinetics during Extracorporeal Membrane Oxygenation (ASAP ECMO) trial show other classes of antimicrobials and sedatives may be equally affected by ECMO, potentially leading to sub-therapeutic drug concentrations if usual dosing regimens are used [146]. Efforts to understand sedative use in patients on ECMO support are regaining interest with the re-emergence of ECMO nowadays.

However, due to its highly lipophilic nature, propofol necessitates being prepared as a lipid emulsion after mixing it with lipid solutions such as glycerol and soybean oil. This latter practice is not recommended by multiple reports in the literature that rejected the use of fat emulsions within ECMO settings to avoid interference with the anticoagulation therapy by increasing the risk of clot formation after fat deposition within the ECMO circuit [153,154]. Therefore, many ICU clinicians avoid its use as part of the sedation regimen during ECMO. Furthermore, as a general note about sedative usage in critically ill patients on ECMO, dosing and monitoring details and efficacy differ largely based on the ECMO modality and reason for ECMO usage. Indeed, sedation needs differ in patients utilizing ECMO as a bridge to transplant compared to those using it for cardiac or respiratory support [130,131]; this is because almost all patients on ECMO as a bridge for transplantation are awake and necessitate light sedation, whereas acute hypoxemic patients require profound sedation and higher sedative dosing in the acute phase [155]. Moreover, longer oxygenator running time and increased risks of oxygenator failure have been reported with extended use of propofol regardless of the underlying setting in all three analyses [131,132,133]. It is also worth noting that sedation methods have always been entirely up to the treating intensivists. Therefore, control of prescribing practices has never been followed. Thus, the effects of the dose received by patients and associated ECMO membrane failure may have been imbalanced [131,156].

It is also important to note that the challenges of opioids and sedatives used in patients on ECMO continue even with ECMO discontinuation when significant dose reduction should take place to counterbalance the rapid decrease in drug Vd. However, although the reduction in dosing may be difficult to calibrate, failure to anticipate such a situation can lead to medication overuse. Patients should then be delicately managed for possible signs of delirium or withdrawal, and the use of a controlled analgosedation approach is necessary [123].

Furthermore, diverse pharmacological and other extracorporeal support, such as renal replacement therapy modalities that have the potential to interact with each other, the increased variability of anticoagulation practice among centers, and the absence of unified guidelines for anticoagulation add to this complexity. Indeed, more complex situations, such as the necessity of concomitant CRRT, build on the need for more robust studies to be performed in the context of ICU patients on ECMO support. Previous studies revealed the difficulties of assigning PK alterations to either ECMO or CRRT in patients requiring both treatment modalities [95]. Further studies with larger patient cohorts are desirable [95]. Nevertheless, many limitations are to be taken into consideration when referring to previously reported studies in this context, such as the single center in nature and the limited number of patients included. Moreover, while some studies described ECMO patients on CRRT including specific parameters such as flow rates (e.g., for blood flow rate, ultrafiltration rate, and effluent flow rate), degrees of hepatic insufficiency to further characterize potential drug clearance effects were not quantified, thus preventing a comprehensive accounting for many of the profound pathophysiological alterations in critically ill patients.

## 6. Conclusions

The discussed evidence-based reports in this document highlight the need for basic and clinical research as a systematic research approach to investigate the multifaceted composite of PK alterations during ECMO support. More advanced efforts to understand the physiochemical properties of the drugs, the etiology, the severity of the underlying illness, and the function of the organs responsible for drug metabolism, as well as ECMO-related factors, would result in a rational interpretation of critically ill patients in the ECMO population relative to PK data.

## Data Availability

Not applicable.
